# Physiological biodistribution of ^18^F-fluciclovine PET in the head: as a baseline for interpretation in patients with suspected glioma

**DOI:** 10.3389/fnume.2026.1850254

**Published:** 2026-06-02

**Authors:** Yumi Abe, Ryogo Minamimoto, Naotoshi Fujita, Marina Higashi, Rintaro Ito, Katsuhiko Kato, Shoichi Deguchi, Fumiharu Ohka, Ryuta Saito, Shinji Naganawa

**Affiliations:** 1 Department of Radiology, Nagoya University Graduate School of Medicine, Nagoya, Aichi, Japan; 2 Department of Integrated Image Information Analysis, Nagoya University Graduate School of Medicine, Nagoya, Aichi, Japan; 3 Department of Radiological Technology, Nagoya University Hospital, Nagoya, Aichi, Japan; 4 Department of Innovative BioMedical Visualization (iBMV), Nagoya University Graduate School of Medicine, Nagoya, Aichi, Japan; 5 Department of Neurosurgery, Nagoya University Graduate School of Medicine, Nagoya, Aichi, Japan

**Keywords:** ^18^F-Fluciclovine, amino acid positron emission tomography, biodistribution, glioma, standardized uptake value

## Abstract

**Introduction:**

Anti-1-amino-3-[^18^F] fluorocyclobutane-1-carboxylic acid (^18^F-fluciclovine; anti-3-[^18^F] FACBC) is an amino acid positron emission tomography (PET) tracer used for the evaluation of brain tumors in cases of suspected or diagnosed glioma. While its uptake characteristics in brain tumors have been reported, systematic data on background uptake in normal-appearing head tissues including extracranial structures remain limited. The purpose of this study was to characterize the background uptake of ^18^F-fluciclovine in intracranial and extracranial head tissues in patients with suspected malignant glioma.

**Methods:**

^18^F-fluciclovine PET/CT images from 20 patients with suspected malignant glioma were retrospectively analyzed. Standardized uptake values (SUVs) were measured in 24 predefined head regions, and inter-regional differences were assessed using one-way ANOVA with Tukey's HSD *post hoc* test. Time–activity curves were generated in 15 patients for dynamic evaluation of temporal uptake patterns. In the overall cohort, 8 patients also underwent ^11^C-methionine PET/CT, and SUVs were compared using paired t-tests and their correlations were assessed using Pearson's correlation coefficient. Statistical significance was set at *P* < 0.05.

**Results:**

^18^F-fluciclovine uptake in the cerebral hemispheres was generally low, with the lowest SUV_mean_ observed in white matter (0.15 ± 0.04). Cortical uptake was uniformly low, with SUV_mean_ and SUV_peak_ of 0.28 ± 0.06 and 0.31 ± 0.07, respectively, and no significant interlobar differences. Among other brain regions, SUV_mean_ was highest in the cerebellar vermis (0.46 ± 0.09), followed by the pons, cerebellum, thalamus/basal ganglia, midbrain, and medulla. Dynamic imaging demonstrated low and stable brain uptake, whereas the pituitary and parotid glands exhibited early peak uptake followed by washout. In patients who also underwent ^11^C-methionine PET, ^11^C-methionine showed significantly higher uptake in all brain regions, whereas ^18^F-fluciclovine was higher in venous structures, skeletal muscle, and the nasal cavity.

**Conclusion:**

This study establishes a detailed baseline of background ^18^F-fluciclovine uptake in normal-appearing intracranial and extracranial head tissues in patients with suspected glioma. These findings may facilitate the distinction between physiological and pathological uptake and inform the interpretation of ^18^F-fluciclovine PET in the assessment of brain tumors.

## 1 Introduction

Anti-1-amino-3-[^18^F]fluorocyclobutane-1-carboxylic acid (^18^F-fluciclovine; anti-3-[^18^F]FACBC) is a synthetic amino acid positron emission tomography (PET) tracer developed for oncological imaging targeting the amino acid transporters highly expressed in tumor cells. The tracer crosses the blood-brain barrier and is primarily taken up by the sodium-dependent alanine–serine–cysteine transporter 2 (ASCT2) and the sodium-independent L-type amino acid transporter 1 (LAT1) (1, 2). Accumulation of ^18^F-fluciclovine is closely associated with the expression levels of these transporters (2, 3). Unlike natural amino acids, ^18^F-fluciclovine is not metabolized intracellularly or incorporated into protein synthesis, allowing for imaging that reflects transporter-dependent accumulation. Although the mechanisms underlying ^18^F-fluciclovine accumulation in tumor tissues have been well studied, reports on background uptake patterns in normal-appearing tissues, particularly in the head, are limited. Notably, ASCT2 is also expressed in various tissues, particularly in cell populations with high metabolic activity (4–8), suggesting that background uptake may be present and potentially influence image interpretation.

Therefore, interpretation of pathological uptake requires a detailed understanding of background uptake patterns in normal-appearing tissues. The aim of this study was to systematically characterize background ^18^F-fluciclovine uptake in intracranial and extracranial head tissues, thereby providing a reference framework to support diagnostic interpretation and the assessment of pathological uptake.

## 2 Material and methods

### 2.1 Patients

This single-center retrospective observational study was performed at Nagoya University Hospital between July 2024 and June 2025. A total of 20 patients were consecutively enrolled during the study period. Patients who underwent ^18^F-fluciclovine positron emission tomography/computed tomography (PET/CT) for suspected malignant glioma were included.

All patients were treatment-naïve at the time of PET/CT, with no history of neurosurgical resection, radiotherapy, or chemotherapy. No patients were receiving corticosteroids, sedatives, or antiepileptic drugs at the time of imaging.

In a subset of patients (*n* = 8), ^11^C-methionine PET/CT was additionally performed. Time-dependent changes in ^18^F-fluciclovine uptake were evaluated using time–activity curves (TACs) in 15 patients with available dynamic data.

This study was approved by the Ethics Review Committee of Nagoya University Hospital (approval number: 2025-206), and the requirement for written informed consent was waived.

### 2.2 PET acquisition protocol

^18^F-fluciclovine was administered intravenously at a mean dose of 230.6 ± 35.8 MBq, and PET acquisition was started 10 min after the administration. Patients were not required to fast prior to PET acquisition, and fasting conditions were not standardized. For pediatric patients, the dose was adjusted according to body weight to match the typical dose used for head ^18^F-fluorodeoxyglucose positron emission computed tomography (FDG-PET/CT) imaging. All PET/CT examinations were performed using Biograph 16 Horizon (Siemens Healthineers, Erlangen, Germany). Imaging was performed in the supine position with the orbitomeatal line positioned horizontally. PET data were acquired in list mode for 20 min, with dynamic images obtained every 2 min, in addition to the 20-min static acquisition. Image reconstruction was performed using the three-dimensional ordered-subsets expectation maximization (3D-OSEM) method (8 iterations, 10 subsets) with CT-based attenuation correction by applying a Gaussian filter with a full width at half maximum of 5 mm, a matrix size of 256 × 256, and a transaxial field of view of 300 mm, following medical guidelines (9) and phantom experiments.

In a subset of patients (*n* = 8), ^11^C-methionine PET/CT was performed based on the clinical indications. Image acquisition was initiated 20 min after tracer injection, followed by a 10-min static scan. Image reconstruction was conducted using the 3D-OSEM method (4 iterations, 10 subsets) with CT-based attenuation correction, a 3-mm Gaussian filter, a matrix size of 512 × 512, and a transaxial field of view of 300 mm.

The interval between the two PET/CT examinations was within 3 weeks for all patients, except one scanned 2 months apart. All patients were included in the analysis after confirmation of stable clinical and neurological status, absence of lesion progression on structural imaging, and no initiation of therapy during the interval between scans.

### 2.3 Image analysis

All image analyses were performed by two nuclear medicine physicians who were board-certified in both radiology and nuclear medicine using SAI Viewer (Fujifilm Medical Co., Tokyo, Japan), which is routinely used in clinical practice. The physiological biodistribution of ^18^F-fluciclovine PET was evaluated using maximum standardized uptake value (SUV_max_) and mean standardized uptake value (SUV_mean_) in 12 intracerebral parenchymal regions (frontal lobe, parietal lobe, temporal lobe, occipital lobe, white matter, thalamus, basal ganglia, midbrain, pons, medulla oblongata, cerebellum, and cerebellar vermis) and in 12 extracerebral regions (pituitary gland, pineal gland, choroid plexus, venous sinus, cavernous sinus, lacrimal gland, parotid gland, pharynx, nasal cavity, muscle, bone marrow, and skin). In this study, “extracerebral” refers to structures outside the brain parenchyma, including intracranial non-parenchymal structures (e.g., the pituitary gland, pineal gland, choroid plexus, venous sinus, and cavernous sinus) as well as extracranial tissues.

**Figure 1:**
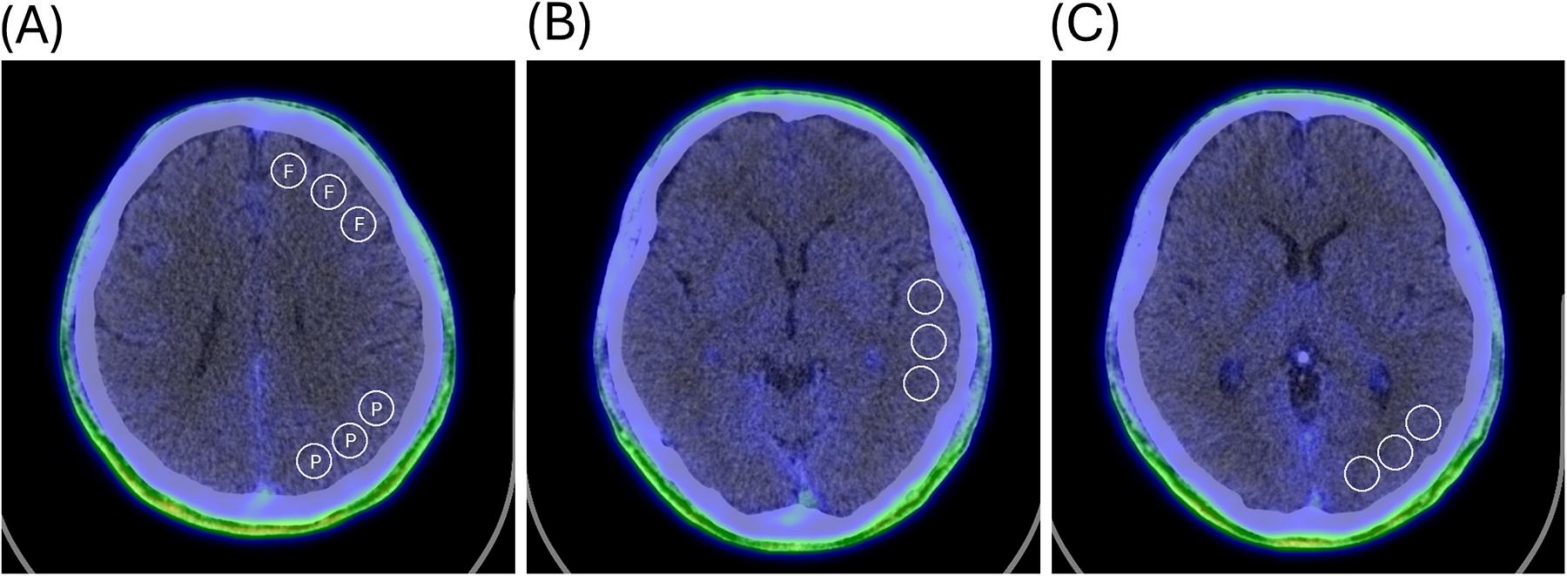
Manual placement of VOIs in each area of the head. Representative VOI placement in contralateral normal-appearing cerebral cortex. Three 1.5-cm spherical VOIs were placed in gray matter corresponding to each lesion for mean SUV measurement. **(A)** Frontal and parietal lobes (F, frontal; P, parietal); **(B)** temporal lobe; **(C)** occipital lobe.

Volumes of interest (VOIs) were manually placed as spherical regions on the axial images, using CT images for anatomical reference. For the cerebral cortex, three 1.5-cm spherical VOIs were placed in tumor-free contralateral gray matter corresponding to each lesion, as shown in Figure 1, and mean SUV was calculated. This standardized approach, commonly used in ^11^C-methionine PET studies, was applied to minimize measurement error and local uptake variability, and to improve inter-subject comparability (10, 11). Peak standardized uptake value (SUV_peak_) was also calculated in the cerebral cortex using the same VOIs and was defined as the highest average SUV within a 1-cm^3^ spherical volume positioned within the VOI. For the white matter, VOIs were placed in the corona radiata. Cases in which tumor presence prevented accurate assessment of uptake in unaffected tissue were excluded. VOIs were carefully placed on the axial, sagittal, and coronal CT images to avoid overlapping with the surrounding structures. All measurement regions were carefully selected to be sufficiently distant from the tumor lesions, showing no PET hyper-uptake or morphological changes based on imaging and clinical follow-up; in the cerebral hemispheres, contralateral tumor-free regions were used.

TACs were generated using MIM Maestro (Cleveland, Ohio, USA) with VOIs placed in the same manner as for the SUV measurements to obtain dynamic uptake over time.

SUV measurements of the ^11^C-methionine PET images were obtained using the same methodology as that applied for ^18^F-fluciclovine.

### 2.4 Statistical analysis

SUVs are presented as mean ± standard deviation (SD). Regional differences were assessed using one-way ANOVA with Tukey's HSD *post hoc* test. Associations with age were assessed using linear regression. SUVs between ^18^F-fluciclovine and ^11^C-methionine were compared using paired t-tests, and their correlations were assessed using Pearson's correlation coefficient. A two-sided *p* < 0.05 was considered statistically significant.

## 3 Results

### 3.1 ^18^F-fluciclovine PET

A total of 20 patients were included (mean age, 49.6 ± 21.5 years; range: 6–77 years; 12 males and 8 females). Fourteen patients had glioma, 2 had primary central nervous system lymphoma (diffuse large B-cell lymphoma), and histological diagnosis was unavailable in 4 patients.

SUVs for each region are presented in Table 1. ^18^F-fluciclovine uptake within the cerebral hemisphere was generally low, with the lowest SUV_mean_ observed in the cerebral white matter (mean SUV ± SD, 0.15 ± 0.04). Cerebral cortical uptake was low (mean SUV_mean_, 0.28 ± 0.06; mean SUV_peak_, 0.31 ± 0.07) and showed no significant differences among the frontal, parietal, temporal, and occipital lobes. SUV_max_ was similarly uniform and low, suggesting minimal nonspecific uptake throughout the cerebral cortex. The SUV_mean_ ratios of the cerebral cortex to the cerebellum were relatively consistent across lobes, ranging from 0.67 to 0.71.

**Table 1:** Mean SUVs of ^18^F-fluciclovine in (A) intracerebral and (B) extracerebral regions.

(A) Intracerebral regions				
	SUV_mean_		SUV_max_	
Region	Mean ± SD	Range	Mean ± SD	Range
Frontal lobe	0.29 ± 0.07	0.21 – 0.40	0.40 ± 0.06	0.31 – 0.49
Parietal lobe	0.28 ± 0.06	0.20 – 0.40	0.41 ± 0.08	0.28 – 0.55
Occipital lobe	0.28 ± 0.06	0.18 – 0.39	0.41 ± 0.09	0.28 – 0.58
White matter	0.15 ± 0.04	0.09 – 0.21	0.19 ± 0.04	0.13 – 0.27
Basal ganglia	0.40 ± 0.09	0.27 – 0.60	0.48 ± 0.11	0.30 – 0.69
Thalamus	0.40 ± 0.11	0.15 – 0.55	0.50 ± 0.11	0.32 – 0.68
Midbrain	0.36 ± 0.07	0.26 – 0.48	0.49 ± 0.09	0.32 – 0.61
Pons	0.43 ± 0.10	0.23 – 0.59	0.57 ± 0.13	0.29 – 0.80
Medulla oblongata	0.35 ± 0.05	0.24 – 0.43	0.45 ± 0.07	0.31 – 0.54
Cerebellum	0.42 ± 0.09	0.29 – 0.60	0.54 ± 0.11	0.38 – 0.76
Cerebellar vermis	0.46 ± 0.09	0.31 – 0.60	0.59 ± 0.11	0.41 – 0.79

(B) Extracerebral regions				
	SUV_mean_		SUV_max_	
Region	Mean ± SD	Range	Mean ± SD	Range
Pituitary gland	2.32 ± 0.38	1.63 – 3.20	2.87 ± 0.50	2.00 – 3.81
Pineal gland	0.49 ± 0.15	0.28 – 0.94	0.64 ± 0.16	0.36 – 1.09
Choroid plexus	0.60 ± 0.16	0.36 – 0.95	0.70 ± 0.19	0.46 – 1.00
Venous sinus	1.22 ± 0.24	0.60 – 1.72	1.41 ± 0.27	0.71 – 1.96
Cavernous sinus	1.37 ± 0.21	0.96 – 1.90	1.52 ± 0.22	1.06 – 2.09
Lacrimal gland	1.35 ± 0.31	0.63 – 1.96	1.48 ± 0.41	0.49 – 2.26
Parotid gland	3.39 ± 0.82	2.20 – 4.64	4.61 ± 1.09	2.68 – 6.63
Pharynx	2.51 ± 0.48	1.74 – 3.54	3.13 ± 0.61	2.17 – 4.10
Nasal cavity	2.30 ± 0.41	1.28 – 2.94	2.91 ± 0.58	1.64 – 3.63
Muscle	2.23 ± 0.55	1.19 – 3.25	2.90 ± 0.84	1.49 – 4.25
Bone marrow	1.99 ± 0.48	1.13 – 2.82	2.43 ± 0.54	1.59 – 3.60
Skin	1.88 ± 0.55	1.14 – 2.81	2.20 ± 0.68	1.30 – 3.80

Values are presented as mean ± standard deviation, with ranges shown below the mean values.

In the remaining brain regions, SUV_mean_ was highest in the cerebellar vermis (0.46 ± 0.09), followed in descending order by the pons (0.43 ± 0.10), cerebellum (0.42 ± 0.09), thalamus (0.40 ± 0.11), basal ganglia (0.40 ± 0.09), midbrain (0.36 ± 0.07), and medulla (0.35 ± 0.05).

Among extracerebral regions, the highest SUV_mean_ was observed in the parotid gland (3.39 ± 0.82), followed by the pharynx (2.51 ± 0.48), pituitary gland (2.32 ± 0.38), nasal cavity (2.30 ± 0.41), muscle (2.23 ± 0.55), bone marrow (1.99 ± 0.48), and skin (1.88 ± 0.55). SUV_max_ showed a similar pattern. In addition, uptake in the muscles, bone marrow, and skin was heterogeneous in distribution. High uptake was observed in the posterior neck, lateral pterygoid, and extraocular muscles, as well as in the bone marrow of the clivus and occipital bone and the vertex skin.

### 3.2 Time-dependent changes of ^18^F-fluciclovine uptake

**Figure 2:**
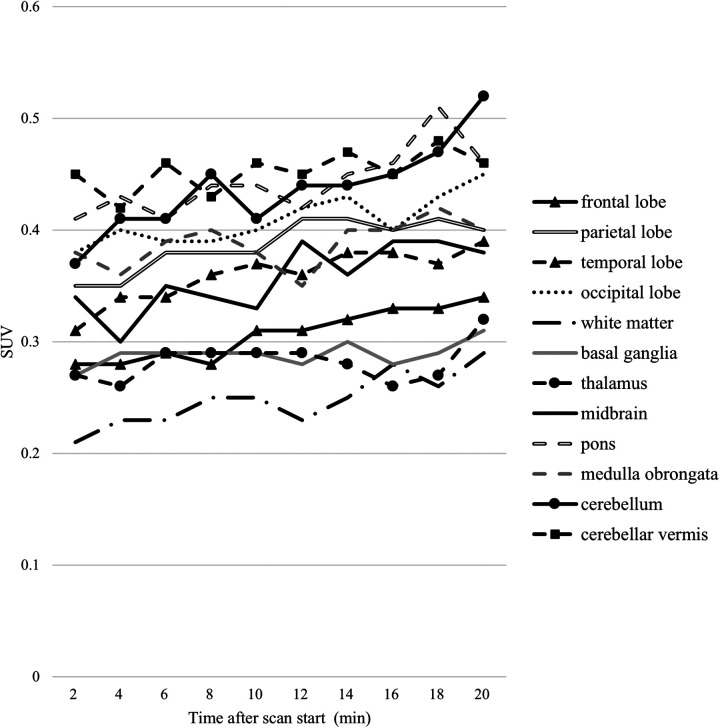
Time-activity curves for intracerebral regions. ^18^F-fluciclovine uptake showed a slight time-dependent increase across all brain regions; however, the absolute differences in SUV remained minimal.

**Figure 3:**
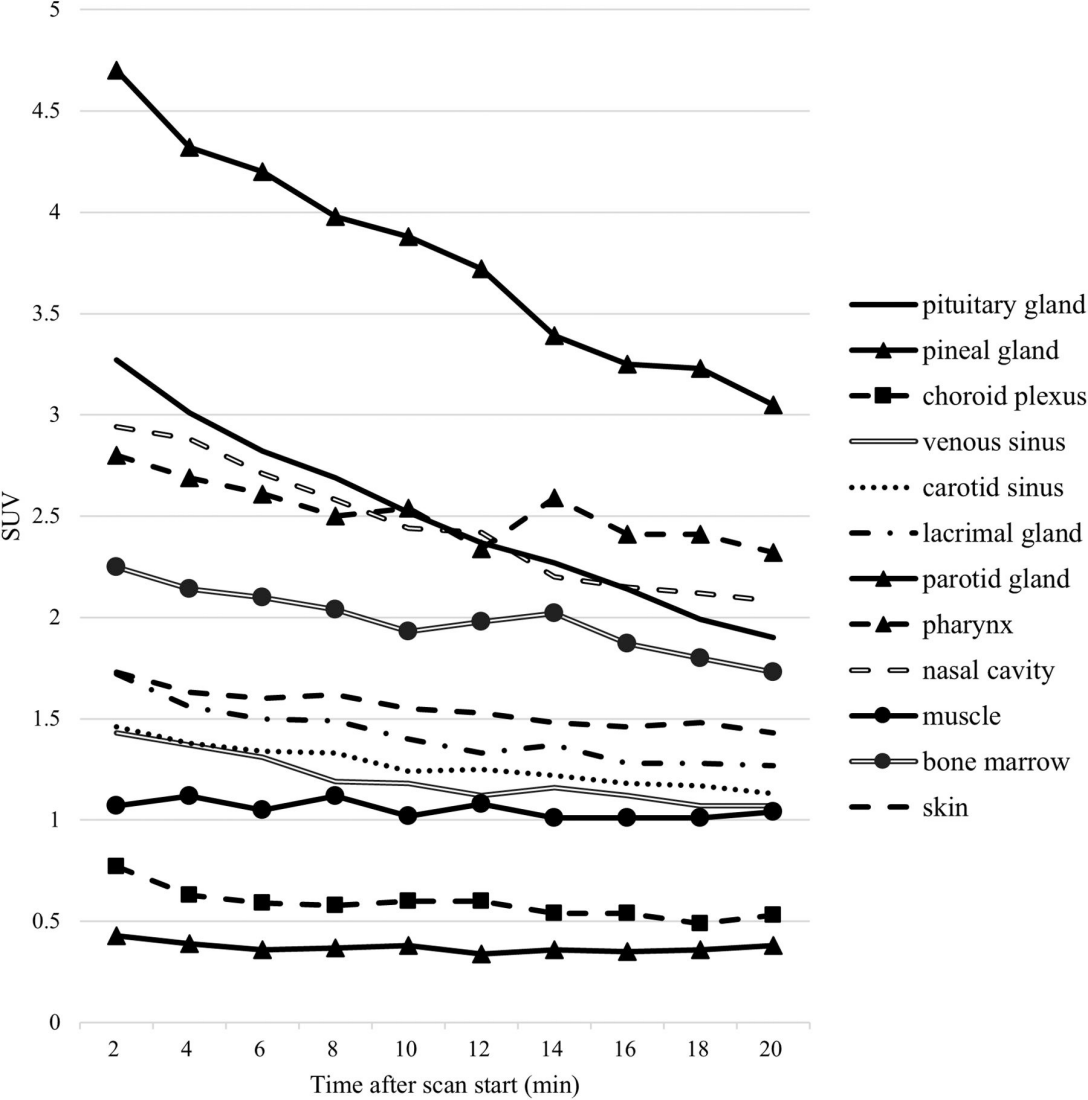
Time-activity curves for extracerebral regions. In the extracerebral regions, ^18^F-fluciclovine uptake in the pituitary and parotid glands peaked at the beginning of dynamic acquisition, followed by a marked decline over time. Gradual decreases were observed in the choroid plexus, venous sinuses, cavernous sinuses, lacrimal glands, pharynx, nasal cavity, and bone marrow.

Among the patients, dynamic data were available for 15 patients (mean age, 55.6 ± 17.7 years; range: 20–77 years; 10 males and 5 females). Dynamic PET imaging was initiated 10 min after intravenous injection of ^18^F-fluciclovine, and time-dependent changes in SUV_mean_ over the subsequent 20 min were analyzed for each region. The regional time–activity curves for intracerebral and extracerebral regions are shown in Figures 2, 3, respectively.

In the brain parenchyma, SUV_mean_ demonstrated slight time-dependent increases across all regions during the 2–20-min acquisition period. In the cerebral cortex, absolute SUV changes were minimal (0.05–0.08) across the frontal, parietal, temporal, and occipital lobes. In other brain regions, the increases ranged from 0.01 (vermis) to 0.15 (cerebellum). Despite relatively large percentage changes in some regions, the absolute SUV differences were small.

In extracerebral regions, the pituitary gland and parotid gland demonstrated the highest SUV at the first 2-min time frame, followed by a marked decline over time (−41.9% and −35.1%, respectively). Moderate decreases (22.6–31.2%) were observed in the choroid plexus, nasal cavity, lacrimal gland, venous sinus, bone marrow, and cavernous sinus.

### 3.3 Age-related changes

Correlations between age and SUVs in the intracerebral regions were not significant. Based on SUV_mean_, the correlation coefficients were minimal (*r* = −0.16–0.20, *p* = 0.1–0.79), indicating negligible associations with age. In extracerebral regions, both the pineal gland and bone marrow demonstrated negative correlations with age (pineal gland: *r* = −0.64, *P* = 0.003; bone marrow: *r* = −0.63, *P* = 0.003). Conversely, the cavernous sinus exhibited a positive correlation with age (*r* = 0.48, *P* = 0.03). Although not statistically significant, several extracerebral regions demonstrated age-related trends in uptake. Uptake in the skin tended to decrease with age (*r* = −0.34, *P* = 0.12), whereas uptake in the muscle, venous sinus, and nasal cavity tended to increase (muscle: *r* = 0.39, *P* = 0.09; venous sinus: *r* = 0.33, *P* = 0.1). Similar trends were also observed for SUV_max_.

### 3.4 ^18^F-fluciclovine compared to ^11^C-methionine PET

Eight of the 20 patients underwent ^11^C-methionine PET/CT (mean age, 41.8 ± 13.6 years; range, 20–62 years; 6 males and 2 females).

Regional SUV_mean_ comparisons in intracerebral and extracerebral regions are provided in Supplementary Table. In all brain regions, SUV_mean_ for ^18^F-fluciclovine was significantly lower than that of ^11^C-methionine (all *p* ≤ 0.004). No significant correlations were observed between the two tracers. A moderate negative correlation was observed in the medulla oblongata (*r* = −0.68); however, this did not reach statistical significance (*p* = 0.07). SUV_peak_ in the cerebral cortex was also significantly higher for ^11^C-methionine than for ^18^F-fluciclovine across all cortical regions (*p* < 0.001), with no significant correlation between the two tracers (Table 2).

**Table 2:** SUV_peak_ comparison and correlation between ^18^F-fluciclovine and ^11^C-methionine in the cerebral cortex.

	SUV_peak_			Correlation	
Region	^18^F-fluciclovine Mean SUV ± SD (range)	^11^C-methionine Mean SUV ± SD (range)	*P* value	Coefficient (*r*)	*P* value
Frontal lobe	0.30 ± 0.06 (0.22–0.38)	1.19 ± 0.21 (0.90–1.42)	< 0.001	0.03	0.94
Parietal lobe	0.30 ± 0.07 (0.22–0.41)	1.23 ± 0.22 (0.87–1.47)	< 0.001	−0.07	0.86
Temporal lobe	0.30 ± 0.06 (0.24–0.40)	1.15 ± 0.20 (0.83–1.37)	< 0.001	−0.14	0.73
Occipital lobe	0.31 ± 0.07 (0.23–0.42)	1.32 ± 0.25 (0.90–1.65)	< 0.001	0.01	0.97

Values are presented as mean ± standard deviation, with ranges shown in parentheses. *P* values indicate comparisons between tracers. Correlation coefficients (*r*) and corresponding *P* values were calculated using Pearson correlation analysis.

In extracerebral regions, SUV_mean_ was higher for ^11^C-methionine than for ^18^F-fluciclovine in the pineal gland, choroid plexus, venous sinus, cavernous sinus, lacrimal gland, nasal cavity, and muscle (all *p* ≤ 0.01). Moderate positive correlations were observed in the muscle and skin (*r* = 0.71, *p* = 0.047 and *r* = 0.71, *p* = 0.049, respectively), whereas no significant correlations were found in the other regions.

### 3.5 Incidental findings

In a patient with neurofibromatosis, a subcutaneous neurofibroma in the cheek demonstrated SUV_max_ of 3.37 and SUV_mean_ of 2.77. An incidentally detected skin nodule in the head demonstrated SUV_max_ of 1.74 and SUV_mean_ of 1.35. In three cases of maxillary sinusitis, mild tracer uptake was observed in mucosal thickening and/or fluid retention (mean SUV_max_, 1.10 [range 0.71–1.56]; mean SUV_mean_, 0.91 [range 0.62–1.30]). Representative images are shown in Supplementary Figure.

## 4 Discussion

We evaluated the background uptake of ^18^F-fluciclovine in the head and analyzed distribution patterns, time-dependent changes, and age-related effects.

^18^F-fluciclovine uptake in brain tissue was generally low, with uniformly low uptake across all regions of the cerebral cortex, indicating minimal non-specific accumulation. Comparison with ^11^C-methionine PET revealed significantly lower uptake of ^18^F-fluciclovine in all brain regions, which is consistent with previous reports (2, 12, 13). The observed differences in tracer uptake were likely due to differences in the distribution and expression characteristics of amino acid transporters. ^11^C-methionine is primarily transported via LAT1 and LAT2, whereas ^18^F-fluciclovine is mainly transported via ASCT2, with limited involvement of LAT1 (14–17). ASCT2 expression in brain endothelial cells has been reported to be lower than that in LAT1 (13), which may partly explain the differences in the uptake of the two tracers. Furthermore, as a non-natural amino acid, ^18^F-fluciclovine is not metabolized intracellularly, which may contribute to its low accumulation in normal brain tissue (12–14). Such low uptake in the normal brain may enhance contrast with the tumor tissue and may be useful in preoperative tumor margin delineation.

Brain uptake increased slightly during dynamic acquisition (2–20 min), with minimal absolute SUV variation (≤ 0.15). This pattern likely reflects progressive tracer delivery and transporter-mediated uptake. Consequently, SUV measurements are unlikely to be substantially influenced by minor timing variations within this interval.

No significant age-related changes were observed in intracerebral uptake. Previous studies using ^11^C-methionine PET have reported inconsistent findings regarding age-related effects. O'Tuama et al. described a marked decrease in brain uptake with increasing age (18), whereas other studies found no significant association between age and uptake (19). These discrepancies may be attributable to differences in imaging protocols, ROI definitions, quantification methods, and study populations.

No significant correlations were observed between ^18^F-fluciclovine and ^11^C-methionine uptake in the brain, which may reflect differences in transporter distribution. However, Michaud et al. reported correlations in the contralateral cerebral hemisphere and cerebellum (13), suggesting that variations in VOI placement, imaging conditions, and study design may influence these findings.

In intracranial structures outside the cerebral cortex, higher uptake was observed in the pituitary gland, cavernous sinus, venous sinus, and choroid plexus than in the cerebral cortex, which is consistent with previous reports (9, 12). This accumulation likely reflects high ASCT2 expression, a relatively permeable blood-brain barrier, and contributions from blood pool activity. In particular, high uptake in the pituitary gland may reflect high metabolic activity associated with hormone synthesis, together with its rich blood supply and lack of an effective blood–brain barrier. ^18^F-fluciclovine uptake in the cavernous and venous sinuses was significantly higher than that of ^11^C-methionine, possibly because ^11^C-methionine is incorporated into protein synthesis and rapidly cleared from the bloodstream, whereas ^18^F-fluciclovine is not, making it more susceptible to blood-pool effects. In contrast, ^11^C-methionine demonstrated significantly higher uptake in the pineal gland and choroid plexus. These differences may reflect variations in amino acid transport pathways and intracellular handling between the two tracers, as well as differences in tracer kinetics. In addition, the pineal gland uptake decreased with age, likely reflecting the age-related phenomenon of pineal gland calcification. This phenomenon increases in prevalence with age (20) and involves the replacement of metabolically active pinealocytes with inactive calcified tissue (21), thereby reducing overall amino acid uptake. This may also reflect decreased local blood flow. Conversely, the cavernous sinus uptake increased with age, likely reflecting age-related changes in metabolic activity, ASCT2 transporter expression in the sinus walls, and blood flow.

Outside the intracranial region, elevated uptake was observed in the parotid gland, pharynx, nasal cavity, muscle, bone marrow, and skin, suggesting a relationship between amino acid metabolism, ASCT2 expression, and regional blood flow. High ASCT2 expression has been reported in metabolically active cell populations such as stem cells (7), which may partly explain the observed uptake.

The parotid glands showed the highest ^18^F-fluciclovine uptake among extracerebral regions, which is in agreement with previous reports describing moderate accumulation (4, 22, 23). Previous ^11^C-methionine PET studies have reported decreased uptake in Sjögren's syndrome (24), suggesting that parotid uptake in ^18^F-fluciclovine PET may reflect metabolically active tissues involved in secretory functions.

Moderate uptake in the nasal cavity and pharynx was also consistent with previous reports (1, 22, 23). The mucosal epithelium of the nasopharynx exhibits high turnover and immune activity, which may contribute to metabolic activity, ASCT2 expression, and abundant blood flow, thereby explaining the observed uptake.

Within the bone marrow, uptake tended to be higher in the clivus and occipital bone. This distribution likely reflects hematopoietic activity and is consistent with prior reports linking ^18^F-fluciclovine and ^11^C-methionine uptake to the presence of red marrow (1, 23). Furthermore, ^18^F-fluciclovine uptake in the bone marrow decreased with age, likely reflecting physiological declines in hematopoietic activity and red marrow volume. In addition, ^18^F-fluciclovine accumulation has been reported in multiple myeloma, bone metastases, and benign lesions such as osteochondromas, Schmorl's nodes, and fractures (1, 22, 23, 25, 26). In contrast, compared with FDG, ^18^F-fluciclovine uptake in the intervertebral discs and facet joints is generally lower (23). When red marrow is sparse or uptake appears relatively intense, ^18^F-fluciclovine accumulation in the bone marrow should be interpreted in conjunction with CT or MRI to avoid overestimation.

Skeletal muscle uptake varied by location, with relatively high accumulation in the posterior neck, lateral pterygoid, and extraocular muscles, consistent with previous studies (1, 27, 28). In addition, skeletal muscle uptake was significantly higher than that of ^11^C-methionine. This pattern likely reflects multiple factors, including muscle composition, functional demand, perfusion, and amino acid transporter expression. Glutamine-mediated inhibition of ^18^F-fluciclovine efflux has also been proposed as a contributing mechanism (27). However, the underlying mechanisms of this heterogeneous uptake remain unclear (28).

The skin uptake was high from the vertex to the occiput, likely reflecting high metabolic activity and transporter expression in stem cells within the basal epidermal layer, hair follicles, and eccrine glands. ^18^F-fluciclovine accumulation has also been reported in malignant skin tumors, including squamous cell carcinoma and melanoma, as well as in inflammatory skin lesions (1, 23, 25, 29). Strong uptake in the skin requires careful interpretation in conjunction with clinical findings to distinguish physiological from pathological uptake. In addition, moderate positive correlations between ^18^F-fluciclovine and ^11^C-methionine were observed in muscle and skin, suggesting partially shared determinants such as perfusion or amino acid transport. However, given the small sample size, these findings should be interpreted cautiously.

Incidental findings included the uptake of neurofibromas, head skin nodules, and sinusitis. Previous studies have similarly reported uptake in benign tumors such as meningiomas and neurogenic tumors (1, 25, 29, 30), as well as in inflammatory lesions (1, 23). These observations indicate that ^18^F-fluciclovine uptake is not tumor-specific and can reflect increased cellular proliferation or metabolic activity in non-neoplastic lesions. Incidental uptake should be interpreted in conjunction with clinical and imaging findings.

Taken together, background ^18^F-fluciclovine uptake appears to be influenced by multiple factors, including amino acid transporter distribution, local metabolism, and blood flow. To our knowledge, this study provides the first comprehensive characterization of background uptake in head tissues. For the assessment of tumor extent in brain tumors, the generally very low uptake throughout the brain and the lack of substantial differences among cortical regions may facilitate the delineation of pathological uptake. In contrast, relatively higher physiological uptake in venous sinuses and bone marrow may confound interpretation, particularly for tumors near the skull base or tentorium, where such uptake may mimic tumor uptake. Awareness of physiological extracranial uptake patterns may help avoid false-positive interpretations.

This study has several limitations. First, participants underwent PET/CT for clinical indications rather than being healthy volunteers; thus, background uptake may not fully represent normal physiology, and the small, heterogeneous cohort with incomplete histological confirmation limits generalizability. In particular, “normal-appearing” tissue in patients with suspected glioma may not reflect true physiological uptake, as subtle metabolic alterations cannot be excluded. Nevertheless, uptake in contralateral non-affected regions was broadly consistent with previous reports (2, 12, 13), supporting the validity of our findings. As the cohort consisted of patients with suspected glioma, the results may reflect routine clinical practice, enhancing clinical applicability. Second, the sample size was relatively small, limiting generalizability and statistical robustness, particularly for comparisons with ^11^C-methionine PET and time–activity analyses. These subgroup analyses should be considered exploratory. In addition, non-uniform intervals between scans may have affected comparisons. Dynamic imaging was initiated at 10 min post-injection; therefore, early tracer kinetics were not assessed. Third, fasting time prior to imaging was not standardized, which may have influenced tracer uptake due to variability in plasma amino acid levels. Fourth, SUV-based semi-quantitative analysis is affected by blood flow, partial volume effects, and measurement variability. VOIs were manually defined, potentially introducing observer variability, and interobserver reproducibility was not assessed, although efforts were made to standardize VOI placement using anatomical landmarks. Furthermore, multiple regional comparisons may increase the risk of type I error despite ANOVA with *post hoc* testing; thus, results should be interpreted with caution. Finally, imaging and analysis were performed using a single PET system and software, and reproducibility across different institutions and scanners remains to be established. Future multicenter studies with larger cohorts and standardized protocols are warranted.

In summary, our findings characterize the physiological distribution of ^18^F-fluciclovine uptake in the head and may aid in distinguishing physiological from pathological uptake, improving diagnostic confidence in clinical PET interpretation. The observed uptake patterns, including extracranial distribution and dynamic behavior, may provide complementary information for lesion assessment and differential diagnosis. Further validation in larger, multicenter cohorts is warranted. ^18^F-fluciclovine PET may have utility in tumor margin delineation and preoperative assessment in brain tumors (31).

## Data Availability

The original contributions presented in the study are included in the article/Supplementary Material, further inquiries can be directed to the corresponding author/s.
