# Supplementary material for: Physiological biodistribution of 18F-fluciclovine PET in the head: as a baseline for interpretation in patients with suspected glioma
Source: Front Nucl Med. 2026 Jun 2;6:1850254. doi: 10.3389/fnume.2026.1850254 (PMC13269264; doi:10.3389/fnume.2026.1850254)
Supplement: Supplementary file 1 [file Datasheet2.pdf]

Supplementary Table

Comparison of regional SUV_{mean} between ^{18}F-fluciclovine PET and ^{11}C-methionine PET in (A) intracerebral and (B) extracerebral regions

(A) Intracerebral regions

	SUV_{mean}			Correlation	
	**^{18}F-fluciclovine Mean SUV ± SD (range)**	**^{11}C-methionine Mean SUV ± SD (range)**	**P value**	**Coefficient (r)**	**P value**
**Frontal lobe**	0.28±0.06 (0.21–0.37)	1.10±0.21 (0.81–1.31)	< 0.001	-0.02	0.96
**Parietal lobe**	0.28±0.06 (0.22–0.39)	1.15±0.23 (0.78–1.40)	< 0.001	0.06	0.89
**Temporal lobe**	0.29±0.05 (0.23–0.38)	1.05±0.18 (0.77–1.23)	< 0.001	-0.21	0.61
**Occipital lobe**	0.28±0.06 (0.22–0.39)	1.22±0.23 (0.81–1.56)	< 0.001	0.08	0.86
**White matter**	0.15±0.04 (0.11–0.21)	0.65±0.11 (0.48–0.80)	< 0.001	-0.11	0.79
**Basal ganglia**	0.39±0.08 (0.31–0.57)	1.41±0.20 (1.01–1.64)	< 0.001	-0.05	0.90
**Thalamus**	0.42±0.09 (0.30–0.55)	1.45±0.26 (1.05–1.77)	< 0.001	-0.26	0.53
**Midbrain**	0.37±0.06 (0.30–0.48)	1.10±0.16 (0.86–1.27)	< 0.001	-0.40	0.32
**Pons**	0.45±0.10 (0.32–0.59)	1.41±0.29 (1.04–1.92)	< 0.001	-0.23	0.59
**Medulla oblongata**	0.35±0.04 (0.25–0.38)	0.84±0.30 (0.45–1.26)	0.004	-0.68	0.07
**Cerebellum**	0.44±0.09 (0.32–0.56)	1.51±0.25 (1.17–1.84)	< 0.001	-0.09	0.83
**Cerebellar vermis**	0.48±0.08 (0.40–0.59)	1.56±0.26 (1.18–1.88)	< 0.001	0.23	0.59

(B) Extracerebral regions

	SUV_{mean}			Correlation	
	^{18}F-fluciclovine Mean SUV ± SD (range)	^{11}C-methionine Mean SUV ± SD (range)	P value	Coefficient (r)	P value
**Pituitary gland**	2.30±0.29 (1.85–2.87)	2.15±0.24 (1.59–2.35)	0.35	-0.20	0.63
**Pineal gland**	0.49±0.09 (0.37–0.66)	1.09±0.22 (0.71–1.42)	< 0.001	0.38	0.35
**Choroid plexus**	0.66±0.14 (0.51–0.88)	1.27±0.46 (0.72–2.21)	0.01	-0.30	0.47
**Venous sinus**	1.24±0.13 (1.12–1.52)	0.95±0.19 (0.68–1.15)	0.002	0.53	0.17
**Cavernous sinus**	1.33±0.10 (1.20–1.46)	1.06±0.12 (0.89–1.22)	< 0.001	0.39	0.33
**Lacrimal gland**	1.35±0.19 (1.01–1.67)	2.26±0.36 (1.63–2.69)	< 0.001	-0.13	0.76
**Parotid gland**	3.51±0.98 (2.20–4.60)	3.78±0.77 (2.87–5.06)	0.39	0.57	0.14
**Pharynx**	2.65±0.44 (2.10–3.54)	2.90±1.08 (1.59–4.81)	0.47	0.53	0.17
**Nasal cavity**	2.40±0.26 (2.11–2.94)	1.89±0.37 (1.49–2.67)	0.01	0.16	0.70
**Muscle**	2.50±0.45 (1.76–3.25)	1.55±0.19 (1.37–1.79)	< 0.001	0.71	0.047
**Bone marrow**	2.27±0.36 (1.80–2.82)	2.09±0.61 (1.59–3.50)	0.38	0.45	0.26
**Skin**	2.18±0.37 (1.51–2.75)	2.21±0.62 (1.71–3.53)	0.85	0.71	0.049

Values are presented as mean ± standard deviation, with ranges shown in parentheses. P values indicate comparisons between tracers. Correlation coefficients (r) and corresponding P values were calculated using Pearson correlation analysis.
